# Mammogram Analysis with YOLO Models on an Affordable Embedded System

**DOI:** 10.3390/cancers18010070

**Published:** 2025-12-25

**Authors:** Anongnat Intasam, Nicholas Piyawattanametha, Yuttachon Promworn, Titipon Jiranantanakorn, Soonthorn Thawornwanchai, Pakpawee Pichayakul, Sarawan Sriwanichwiphat, Somchai Thanasitthichai, Sirihattaya Khwayotha, Methininat Lertkowit, Nucharee Phakwapee, Aniwat Juhong, Wibool Piyawattanametha

**Affiliations:** 1 Department of Biomedical Engineering, School of Engineering, King Mongkut’s Institute of Technology Ladkrabang, Ladkrabang, Bangkok 10520, Thailand; anongnat.in@kmitl.ac.th (A.I.);; 2 School of Engineering, Michigan State University, East Lansing, MI 48823, USA; 3 Diagnostic Radiology and Nuclear Medicine Department, The National Cancer Institute, Ratchatewi, Bangkok 10400, Thailand; 4 Udon Thani Cancer Hospital, Muang, Udon Thani 41300, Thailand; 5 Department of Electrical and Computer Engineering, Michigan State University, East Lansing, MI 48823, USA; 6 Institute for Quantitative Health Science and Engineering, Michigan State University, East Lansing, MI 48823, USA

**Keywords:** breast cancer diagnosis, object detection, mammogram, YOLOv5, YOLOv8, YOLOv10, YOLOv11, computer-aided detection, artificial intelligence, CAD

## Abstract

Background/Objectives: Breast cancer persists as a leading cause of female mortality globally. Mammograms are a key screening tool for early detection, although many resource-limited hospitals lack access to skilled radiologists and advanced diagnostic tools. Deep learning-based computer-aided detection (CAD) systems can assist radiologists by automating lesion detection and classification. This study investigates the performance of various You Only Look Once (YOLO) models and a Hybrid Convolutional-Transformer Architecture (YOLOv5, YOLOv8, YOLOv10, YOLOv11, and Real-Time-DEtection Transformer (RT-DETR)) for detecting mammographic lesions on an affordable embedded system. Methods: We developed a custom web-based annotation tool to enhance mammogram labeling accuracy, using a dataset of 3169 patients from Thailand and expert annotations from three radiologists. Lesions were classified into six categories: Masses Benign (MB), Calcifications Benign (CB), Associated Features Benign (AFB), Masses Malignant (MM), Calcifications Malignant (CM), and Associated Features Malignant (AFM). Results: Our results show that the YOLOv11n model is the optimal choice for the NVIDIA Jetson Nano, achieving an accuracy of 0.86 and an inference speed of 6.16 ± 0.31 frames per second. A comparative analysis with a graphics processing unit (GPU)-powered system revealed that the Jetson Nano achieves comparable detection performance at a fraction of the cost. Conclusions: The current research landscape has not yet integrated advanced YOLO versions for embedded deployment in mammography. This method could facilitate screening in clinics without high-end workstations, demonstrating the feasibility of deploying CAD systems in low-resource environments and underscoring its potential for real-world clinical applications.

## Simple Summary

Breast cancer is a leading cause of death among women worldwide, and mammograms are a crucial tool for early detection. However, many resource-limited hospitals face challenges in accessing skilled radiologists and advanced diagnostic systems. This study evaluates the use of various You Only Look Once (YOLO) models, including YOLOv5, YOLOv8, YOLOv10, YOLOv11, and Real-Time-DEtection TRansformer (RT-DETR), for automated mammographic lesion detection on affordable embedded systems, such as the NVIDIA Jetson Nano (NVIDIA Corp., Santa Clara, California, USA). The results demonstrate that the YOLOv11n model performs optimally on this low-cost hardware, achieving an accuracy of 0.86 and an inference speed of 6.16 ± 0.31 frames per second. This research shows that deep learning-based computer-aided detection (CAD) systems can be deployed in low-resource clinical settings, improving access to early breast cancer detection in underserved regions.

## 1. Introduction

Breast cancer is one of the deadliest diseases among women worldwide. In 2024, the United States had 310,720 reported cases of breast cancer, with around 42,250 people dying due to this disease [1]. Breast cancer generally presents no symptoms when it is tiny and treatable. Therefore, breast cancer screening is vital for early detection. According to published data, the long-term survival rate for patients diagnosed with breast cancer is roughly 91% following a five-year assessment period. This underscores the significance of early-stage breast cancer diagnosis, specifically focusing on lesion detection. The difficulty in detecting breast cancer lesions stems from their variety in shape, size, and appearance. Lesions vary in shape and size and might be uneven, spiculated masses, or spherical, and they are often associated with breast cancer. Currently, manual mammogram examinations by radiologists are fraught with challenges that hinder accuracy and efficiency. These challenges include the overwhelming volume of mammograms that must be reviewed daily, which can lead to fatigue and decreased concentration. Furthermore, the shortage of highly experienced radiologists exacerbates these difficulties. As a result, the diagnostic process is often delayed, and there is an increased risk of both false positives and false negatives [2]. Deep learning-based Computer-Aided Diagnosis (CAD) systems have demonstrated excellent performance in automated lesion detection, primarily to reduce radiologists’ workloads and improve screening efficiency. Traditional image processing techniques, such as Gray-Level Co-occurrence Matrix (GLCM), Principal Component Analysis (PCA), and Hough transform, rely on handcrafted feature extraction, which limits their ability to generalize across datasets. State-of-the-art deep learning models, particularly Convolutional Neural Networks (CNNs), have exceptional performance in medical image analysis, including mammography. Among CNN-based object detection architectures, You Only Look Once (YOLO) [3] has gained popularity for real-time applications due to its high speed and accuracy. Unlike two-stage detectors (e.g., faster Regional-Based Convolutional Neural Network), which generate region proposals, YOLO is a single-stage detector, making it significantly faster. Recent versions of YOLOv5–v12 [4,5,6,7,8,9,10] have further optimized network depth, feature aggregation, and computational efficiency, making them well-suited for real-time mammogram analysis. However, to push performance boundaries even further, many modern deep learning models, including some advanced YOLO architectures, have begun to incorporate Transformer-based and hybrid architectures. Transformer-based and hybrid architectures have now become a core technology in Modern deep learning models. These architectures are now crucial for text, computer vision, video, and audio. In Natural Language Processing (NLP), the Transformer is the foundational architecture for Large Language Models (LLMs). Models such as Generative Pre-trained Transformer (GPT) [11,12], Gemini [13,14], Claude [15], Large Language Model Meta AI (LLaMa) [16,17], Pathways Language Model (PaLM) [18,19], and Text-to-Text Transfer Transformer (T5) [20]. The performance models are most likely to recognize, evaluate, and produce text that resembles human perception. The Transformer architecture [21] was initially proposed by Google researchers in 2017 for computer vision tasks optimized for images, including Vision Transformer (ViT) [22], Swin Transformer [23], and DETR [24]. In addition, Transformer-based models are versatile and can be used for text, vision, and audio data, such as the Bootstrapping Language-Image Pre-training (BLIP) [25], Flamingo [26], Whisper [27], and the Segment Anything Model (SAM) [28].

In 2022–2025, transformer-based models have increasingly demonstrated superior performance in mammographic breast cancer detection [29,30,31]. Vision Transformer applied to four-view mammography achieved an Area Under the Curve (AUC) of 0.818 [31], and comparative studies showed that Transformer variants, including ViT, Swin Transformer, and Pyramid Vision Transformer (PVT), performed similarly. The transformer model outperformed leading CNN models, with the best-performing transformer achieving an AUC of 1.0, compared to 0.95 for Residual Network (ResNet) 50 [32]. Integrating Transformers into detection frameworks such as YOLOv4 further improved breast mass analysis [33], while Multiview evaluations confirmed that transformer architectures outperform both CNNs and graph-based models [34]. Transformers have enabled effective multimodal fusion of radiomics, deep imaging features, and Ribonucleic Acid Sequencing (RNA-Seq) biomarkers for high-accuracy Breast Imaging Reporting and Data System (BI-RADS) classification [35]. Hybrid approaches, such as Hybrid Model Network (HybMNet), combine self-supervised Swin Transformer pretraining with CNN fine-grained extraction, achieving strong AUC performance and showing promise for weakly supervised microcalcification detection [36]. Collectively, these studies highlight the growing dominance of Transformer-based and hybrid architectures over traditional CNNs in mammography analysis. For these models to be widely implemented in practice, ViT inherently demands substantial Graphics Processing Unit (GPU) memory, high-performance computing resources, and protracted training durations. As a result, crafting adequately lightweight architectural variants poses a significant challenge for clinical deployment, especially across resource-limited hospitals and community healthcare facilities.

This research was inspired by the lightweight architecture of YOLO and a Hybrid Convolutional–Transformer Architecture for detecting various types, with the goal of enhancing breast cancer screening by analyzing mammogram images. Breast lesions are critical in breast cancer screening and guiding appropriate treatment by professional radiologists. There are three main types of breast lesions: masses, calcifications, and associated features.

Breast masses refer to abnormal lumps or tissue growths detected in the breast. The shape and margin of the mass are critical appearance criteria for distinguishing between benign and malignant masses. A spiculated margin raises the possibility of cancer in the [37]. Breast calcifications are accumulations of calcium inside the breast tissue. Microcalcifications and macrocalcifications are two types of calcifications. Macrocalcifications appear as large white spots randomly distributed throughout the breasts and are usually harmless, requiring no further imaging. Conversely, microcalcifications have been strongly connected to an elevated risk of breast cancer [38]. The distribution of microcalcifications is an essential factor in breast cancer screening and diagnosis. A linear distribution or path within a duct indicates a predicted cancer risk of around 60% [39].

Other features related to breast abnormalities are referred to as Associated Features (AF). These include skin retraction, nipple retraction, skin thickening, trabecular thickening, axillary lymphadenopathy, and architectural distortion. Visualizing these lesions with a mammogram is the basis of the doctor’s treatment and may affect the development of breast cancer. This research focused on axillary lymphadenopathy, nipple retraction, and skin thickening. Primarily, axillary lymphadenopathy refers to the distension of lymph nodes in the axilla, often located in the armpit area. This disorder can have various causes, both benign and malignant. The lymph nodes are part of the lymphatic system, which plays a crucial role in both immune defense and immune function. It usually suggests the body is responding to an underlying condition when it grows swollen or enlarged. Then, nipple retraction, also known as an inverted nipple, occurs when the nipple turns inward into the breast rather than outward. The appearance of a nipple ought to be examined regularly by a healthcare practitioner, as it can suggest underlying medical issues, such as breast cancer. Moreover, skin thickening in the breast is an alteration that causes one part of the breast to appear harder or thicker than the rest of the breast tissue. A variety of conditions, such as inflammatory breast cancer, mastitis, or benign breast diseases, can cause skin thickening in the breast.

The combination of the YOLO model and the NVIDIA Jetson Nano for mammogram screening represents a significant innovation. It brings real-time artificial intelligence (AI)-powered computer vision to low-cost, low-power embedded systems, enabling edge intelligence in resource-constrained environments and providing inexpensive, energy-efficient AI to remote clinics where conventional medical equipment might not be available or too costly. There is a marked scarcity of research applying YOLO to real-time mammogram analysis, especially on accessible, low-cost hardware such as NVIDIA Jetson Nano. The current research landscape is dominated by studies requiring powerful, high-end workstations, effectively excluding clinics and medical units with limited resources. By filling that crucial gap, this research hopes to make AI-powered diagnostic tools more widely available and democratic.

This study evaluates responding to the real-time performance of multiple YOLO versions and a Hybrid Convolutional–Transformer Architecture for mammogram lesion detection, specifically for deployment on embedded hardware (NVIDIA Jetson Nano). The objectives of this study are:

1. We create a mammography dataset (a web-based annotation tool) and train lightweight versions of YOLO and a Hybrid Convolutional–Transformer Architecture (YOLOv5, YOLOv8, YOLOv10, YOLOv11, and RT-DETR) for breast lesion detection, providing a comprehensive performance comparison.
2. We deploy the optimized model on a NVIDIA Jetson Nano, demonstrating real-time inference capability on the device.
3. We discuss the feasibility of this low-cost system for use in resource-limited clinical settings.

The structure of this paper is as follows: Section 2 provides an overview of the YOLO model’s evolution. Section 3 details the methodology employed in this research. Section 4 presents the findings and offers a comprehensive discussion. Finally, Section 5 concludes the overall work presented.

## 2. Related Work

### 2.1. CAD Systems for Mammogram Detection

YOLO has played a role, driving leapfrog advancements in CAD system development, with ongoing research aimed at breaking previous limitations to achieve maximum efficiency in lesion detection. For instance, Al-Masni et al. proposed using YOLO for CAD to detect masses in mammograms. The system performs well even in challenging cases, such as masses located over the pectoral muscles or within dense tissue regions [40]. Subsequently, Aly et al. employed YOLOv1, YOLOv2, and YOLOv3 to classify breast masses, using K-means clustering to generate anchor boxes for training on mammograms. Their findings indicated that YOLOv3 outperformed the other versions in terms of detection and classification accuracy [41]. Currently, many research efforts have pivoted to using new-generation YOLO models and high-performance detectors to achieve more accurate results. One study successfully developed a YOLOv5 model that uses the Eigen-CAM solution to effectively highlight malignant lesions [42].

Furthermore, in 2024, Shia et al. evaluated the performance of the advanced YOLOv8 model for detecting microcalcifications in the breast, a critical early warning sign of breast cancer [43]. Since breast cancer is a complex condition that can manifest in numerous ways, its symptoms are not limited to just a palpable lump. We acknowledge the critical need to detect multiple types of breast abnormalities; a powerful analytical tool is required. The NVIDIA Jetson is an ideal choice for running AI programs and serves as a low-cost, high-performance engine for deploying models capable of detecting these diversities. This facilitates timely intervention, drastically improves patient outcomes, and represents a tangible step toward achieving healthcare equity by ensuring that a person’s geographic location does not determine their chance of survival. For example, this research [44] successfully developed a real-time skin cancer detection system on the NVIDIA Jetson using a highly accurate YOLO model, demonstrating particular effectiveness against dangerous melanoma. Another research team presented a handheld device for diagnosing lung disease, powered by an NVIDIA Jetson Nano and a ResNet-50 AI model [45]. The system analyzes Chest X-ray (CXR) images in real-time to classify them as coronavirus disease 2019 (COVID-19), Tuberculosis, or normal with a high accuracy of 98.79%.

### 2.2. Challenges of the YOLO Model Until the Present

**Figure 1:**
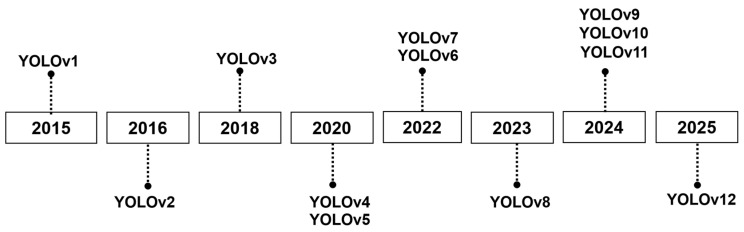
The evolution timeline of YOLO models from 2015 to the present.

YOLO is a model for predicting bounding boxes with confidence scores and image classes. The YOLO family evolved from 2015 to 2025, as depicted in Figure 1. The principal YOLO models include YOLOv1 [3], YOLOv2 [45], YOLOv3 [46], YOLOv4 [47], YOLOv5, YOLOv6 [4], YOLOv7 [5], YOLOv8, YOLOv10 [7], YOLOv11 [8], and YOLOv12 [9].

In 2016, Joseph Redmon and a group of researchers developed the YOLOv1 model. The YOLOv1 model was motivated by the GoogLeNet model [48]. This model is an object detection model that performs one-stage detection without an intermediate region proposal stage. It works by dividing images into grid cells to detect objects within the image. Each grid cell represents a bounding box and is associated with confidence probabilities. The confidence probabilities must be zero if nothing is detected within a cell. YOLOv2, also known as YOLO9000, was introduced a year later by the Allen Institute for AI and the University of Washington. YOLOv2 used Darknet-19 as the backbone feature-extraction network, consisting of 19 convolutional and max-pooling (MP) layers.

Additionally, YOLOv2 implemented a multi-scale training strategy, in which the network was trained on images of varying resolutions at each iteration. This approach allowed the model to handle objects of different sizes effectively. In 2018, the team that developed earlier versions of YOLO developed YOLOv3. Darknet19 was enhanced and renamed Darknet53. Darknet53 comprises 53 convolutional layers with varying filter sizes and strides. The Darknet53 concept is influenced by the ResNet architecture, particularly its use of residual connections [49]. Residual blocks can enable the network to learn and propagate gradients more efficiently. It aids training and enhances the model’s ability to accurately recognize complex features. YOLOv3 has three detection scales for multi-scale detection, whereas YOLOv2 has only two. YOLOv4 and YOLOv5 were released in the following two years. YOLOv4 was developed in 2020 by researchers at the Institute of Information Science at Academia Sinica in Taiwan. YOLOv4’s backbone is a modified version of the Darknet architecture, called Cross-Stage Partial (CSP) Darknet. It integrates the CSP Connections module with the Darknet53 architecture to enhance accuracy and speed.

YOLOv4 incorporates several advanced components, including a Path Aggregation Network (PAN) for multi-scale feature fusion and efficient information flow, a Spatial Pyramid Pooling (SPP) module for capturing multi-scale contextual information, and a Spatial Attention Module (SAM) for enhanced spatial modeling. YOLOv4 is more complex and computationally demanding than YOLOv3 due to its improved architecture, advanced techniques, and optimizations. YOLOv4 requires more computational resources and memory to perform optimally.

**Figure 2:**
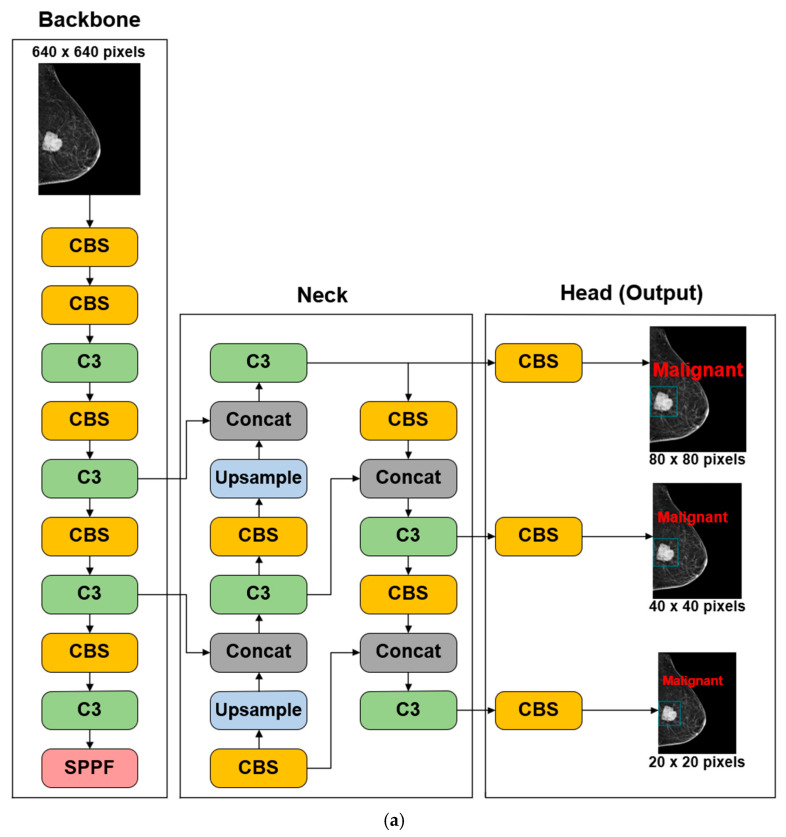

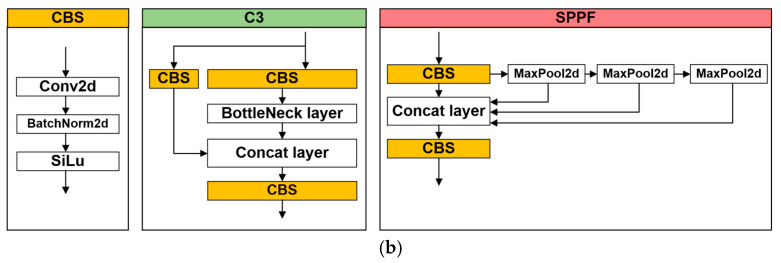
Diagram of the YOLOv5 architecture. (**a**) Backbone, Neck, and Output networks with key components (CBS in yellow, C3 in green, SPPF in red, Concat in grey, and Upsample in light blue colors) in YOLOv5. (**b**) Details of CBS, C3, and SPPF used in the networks of YOLOv5.

YOLOv5, developed and maintained by Ultralytics since 2020, employs a modified backbone architecture tailored for efficient object detection. Each convolutional block consists of a convolution layer, a batch normalization layer, and a Sigmoid Linear Unit (SiLU) activation function. These convolutional blocks (CBS) serve as the fundamental units for feature extraction. A key characteristic of YOLOv5 is the use of the Cross Stage Partial Bottleneck with three connections (C3) together with a Spatial Pyramid Pooling–Fast (SPPF) layer. The C3 module is derived from the Cross Stage Partial Network (CSPNet) architecture [50], which partitions the feature maps of a base layer into two paths that are later recombined through a cross-stage hierarchy, effectively splitting the gradient flow across different network routes. This design enriches the diversity of gradient information, mitigates redundant gradient propagation, and substantially reduces computational cost. As a result, CSPNet-based detectors achieve faster inference times while maintaining or improving accuracy relative to conventional backbones. The SPPF module is an efficient variant of the original Spatial Pyramid Pooling layer, designed to capture multi-scale contextual information with reduced computation by using repeated max-pooling operations. The overall architecture of the YOLOv5 model is illustrated in Figure 2. Figure 2a illustrates the overall pipeline: the backbone extracts hierarchical features, the neck fuses multi-scale representations through upsampling and concatenation, and the detection head generates predictions at three resolutions (80 × 80, 40 × 40, and 20 × 20) to support detection of objects at different scales. Figure 2b presents the core modules used throughout the network: CBS, C3, and SPPF.

Meituan Technical Team developed YOLOv6. The primary purpose of YOLOv6 is to support industrial tasks. The re-parameterized visual geometry group (RepVGG) [51] or the Efficient Re-parameterized (EfficientRep) serves as the backbone for feature extraction in this model. The Re-parameterized Block (RepBlock) is an essential component of RepVGG. RepBlok is blocked in a small network. In the neck, YOLOv6 uses the PAN [50] to combine feature maps from multiple layers or stages of the network and aggregate them into a single high-resolution feature map. Next, YOLOv7 was introduced in July 2022 by the same researchers who developed YOLOv4. This model relates to reparameterization and model-scaling methods. The re-parameter method combines multiple computational modules into a single module. The model-level and module-level ensembles are two groups that comprise the reparameterization method. One approach is to train various identical models on distinct data and then average their weights. The other approach calculates the average of the model’s weights as a function of the iteration number.

On the other hand, a module-level ensemble divides a module into several identical or various module subdivisions. During inference, several subdivision modules are combined into a corresponding module while training. Model scaling is a technique for scaling an existing model up or down to suit diverse computing devices. The internal structure of YOLOv7, specifically the Extended Efficient Layer Aggregation Network (E-ELAN), serves as the backbone for extracting meaningful features from the input. E-ELAN modified the computation block model to gain a deeper understanding of various features. In addition, YOLOv7 features model scaling, allowing the adaptation of specific model attributes and the generation of models at multiple scales to meet different inference speed requirements.

Recently, YOLOv8 was introduced and is built on the same core as YOLOv5. The backbone and head section of YOLOv8 utilizes the Cross-Stage Partial with 2 Fusion connections (C2f) module to replace the C3 module, which includes the CBS, Split, BottleNeck, and Concat layers. The C2f operation is frequently used to combine higher and lower-resolution feature maps. This enables the model to extract fine-grained details from high-resolution feature maps and contextual information from low-resolution ones. The model can leverage multi-scale information to improve object detection performance by concatenating the feature maps. Overall, the C2f operation is crucial in integrating feature maps of varying resolutions and spatial sizes into concatenation-based object detection models, enabling them to handle objects of varying scales effectively. In summary, CSPDarknet serves as the backbone for both YOLOv5 and YOLOv8, whereas YOLOv7 utilizes its own unique YOLOv7 backbone.

In 2024, the YOLOv10 [7] model was developed to enhance speed, accuracy, and efficiency. This work proposed a Non-Maximum Suppression (NMS)-free training technique for models on double-label tasks with effective corresponding statistics, combining One-to-Many and One-to-One heads to improve training. The One-to-Many Head returns multiple potential matches, while the One-to-One Head selects the best match for inference, eliminating the need for NMS. This model has a Consistent Match Metric to help ensure the best label assignments by considering confidence scores and IoU values. Then, YOLOv11 is the latest advancement in the YOLO family of models. SPPF, Cross-Stage Partial with Spatial Attention (C2PSA), and the Cross Stage Partial Bottleneck with kernel size 2 (C3k2) block are essential components of YOLOv11. The SPPF block efficiently extracts multi-scale features while preserving spatial details. The C2PSA block enhances feature learning by focusing on important image regions. Lastly, the C3k2 optimizes feature aggregation, improving accuracy while maintaining a lightweight model. The latest model, YOLOv12, released in 2025, emphasizes the importance of attention mechanisms. The core of YOLOv12 is Area Attention and Residual Efficient Layer Aggregation Networks (R-ELAN). These developments make it possible to detect small, obscured things. The backbones of the YOLO and RT-DETR models are summarized in Table 1.

## 3. Materials and Methods

### 3.1. Graphical User Interface of the Custom Web-Based Labeling Tool

Labeling tools are crucial in deep learning tasks, such as computer vision, as they enable the accurate representation of features of interest. To annotate mammogram images in this study, we designed a web-based, custom labeling tool tailored to enhance ease of Use for radiologists. This tool was developed by a team at the Advanced Imaging Research Centre (AIRC) at the School of Engineering, King Mongkut’s Institute of Technology, Ladkrabang (KMITL), Bangkok, Thailand, utilizing Hypertext Markup Language (HTML), JavaScript, and Hypertext Preprocessor (PHP).

**Figure 3:**
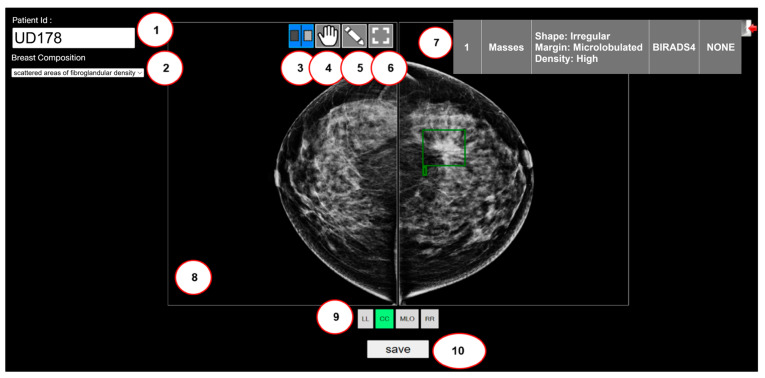
Our custom web-based image annotation labeling tool uses the BI-RADS classification system. The green box (a mass feature) labelled by a radiologist.

The tool features capabilities such as bounding box creation, text annotation, and image zoom. Annotations yield coordinates and dimensions of the lesions, resulting in bounding boxes that detail the mammogram’s lesion types and locations. These annotations are exported and stored in Comma-Separated Values (CSV) format, where each line describes an object and specifies the lesion’s spatial location using four integer coordinates (x1, y1, x2, and y2). These are subsequently translated into the YOLO format. Figure 3 showcases the interface of our custom web-based labeling tool, delineating its primary functions, which include:

1. Patient ID listing,
2. Listing of breast compositions, which enumerate four breast densities: almost entirely fatty, scattered areas of fibroglandular density, heterogeneously dense, and extremely dense,
3. Scrolling feature for individual breasts,
4. Scrolling feature for dual breasts,
5. Creating a bounding box,
6. Resetting the image to its original view,
7. Displaying the lesion type in detail,
8. Displaying the mammograms,
9. Quadruple view selection (CC: Craniocaudal, MLO: Mediolateral Oblique, LL: Left–Left, and RR: Right–Right) of the mammograms, and
10. Saving annotated data.

Furthermore, after post-annotation using the function (5), the web-based tool can delineate eight specific lesion types: Associated Features, Masses, Calcifications, Asymmetries, Architectural Distortion, Intramammary Lymph Nodes, Skin Lesions, and Solitary Dilated Ducts. The “Mass” category emphasizes specific details of shape, margin, and density, while the “Calcification” category provides insights into classifications such as typically benign, suspicious morphology, and distribution patterns.

### 3.2. Mammogram Dataset

#### 3.2.1. Private Dataset

The mammograms used in this study were sourced from a retrospective analysis of 3169 female patients who underwent examinations at the National Cancer Institute, Bangkok, Thailand, and Udon Thani Cancer Hospital, Udon Thani, Thailand. A total of four mammograms were collected per patient, consisting of two standard projection views (Craniocaudal (CC) and Mediolateral Oblique (MLO)) for each breast. The Traditional dataset split was performed strictly on a patient-wise basis to ensure the robustness and clinical validity of the reported results. The total dataset was partitioned into two distinct subsets: 70% (2078 patients) was allocated for model training, and the remaining 30% (891 patients) was reserved as an independent set for performance evaluation (validation).

Furthermore, in the final step of lesion abnormality identification, an additional dataset of 100 patients with benign images and 100 patients with malignant images, not used for training or validation, was used as the test set. This dataset constitutes the final holdout set, extracted specifically to ensure complete independence from the training and validation data. We aimed to assess their accuracy, reliability, and practicality in diagnosing or classifying conditions, which is crucial for their potential use in healthcare settings.

**Figure 4:**
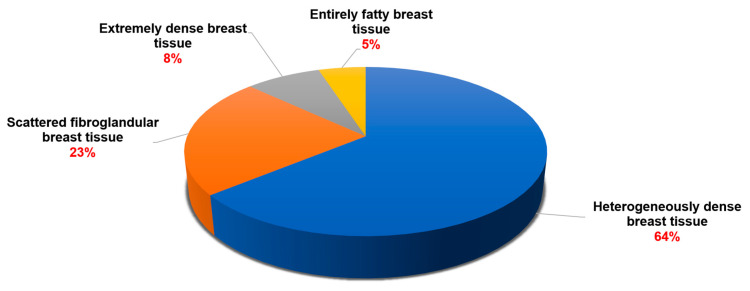
The percentage of dense breast tissue in 3169 patients.

The original mammograms, in two sizes—3328 × 4096 pixels and 2560 × 3328 pixels—were first converted from the Digital Imaging and Communications in Medicine (DICOM) format to Joint Photographic Experts Group (JPEG) before being uploaded to our custom web-based labeling tool. All image data is de-identified using an anonymization function before being retrieved from the Picture Archiving and Communication System (PACS). We exclusively store mammogram images stripped of all patient identifiers. 64% of mammograms depict breasts with heterogeneously dense tissue, while 23% show scattered fibroglandular tissue. Highly dense breast tissue accounts for 8%, and entirely fatty breast tissue makes up about 5%. Figure 4 illustrates the distribution of breast tissue density in all patients. Three radiologists, each with over ten years of experience in breast mammography, meticulously annotated the dataset. This annotation process included a rigorous double-reading procedure to ensure accuracy and completeness, utilizing mammography records from prior screenings or examinations. There are 965 benign images and 506 malignant images. Of the total images, 1471 were selected based on their lesion coordinates (Table 2).

**Figure 5:**
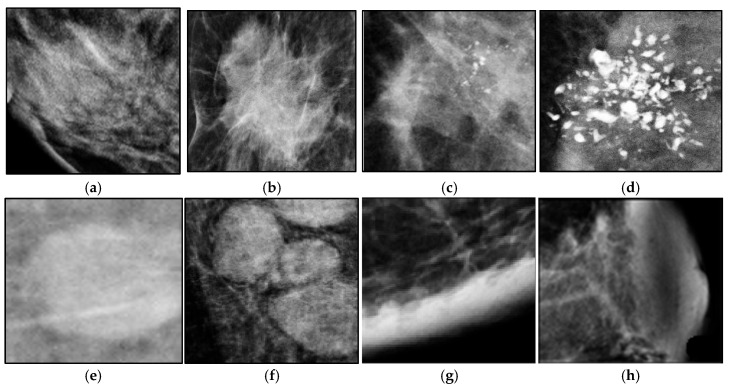
Examples of lesions in mammograms: (**a**) MB lesion, (**b**) MM lesion, (**c**) CB lesion, (**d**) CM lesion, (**e**) AFB lesion (axillary adenopathy), (**f**) AFM lesion (axillary adenopathy), (**g**) AFM lesion (skin thickening), and (**h**) AFM lesion (nipple retraction).

Then, these 1471 images were categorized into six distinct classes: (1) Masses Benign (MB), (2) Masses Malignant (MM), (3) Calcifications Benign (CB), (4) Calcifications Malignant (CM), (5) Associated Features Benign (AFB), and (6) Associated Features Malignant (AFM). The total count of labeled lesions is 1958, distributed as 565 MB lesions, 413 MM lesions, 412 CB lesions, 143 CM lesions, 310 AFB lesions, and 115 AFM lesions. All annotated lesions were utilized in the experiments for this study. Figure 5 provides an example of the annotated lesions. Figure 5a shows an MB lesion, Figure 5b an MM lesion, Figure 5c a CB lesion, and Figure 5d a CM lesion. Figure 5e,f illustrate AFB and AFM lesions associated with axillary adenopathy, respectively, while Figure 5g,h show AFM lesions characterized by skin thickening and nipple retraction, respectively. The YOLO models are trained and validated on the dataset mentioned above to identify six lesions in mammograms.

#### 3.2.2. VinDr-Mammo Dataset

We used Vietnam’s Big Data Research VinDr-Mammo dataset [52]. This dataset is a large-scale mammography designed to enable research on computer-aided diagnosis, specifically for the identification and evaluation of breast cancer using AI and deep learning. A total of 1093 mammograms were used in this study. There are 588 benign images and 505 malignant images.

The dataset was strategically partitioned into three subsets: 70% for training, 20% for validation, and 10% for testing.

The objective of this research is to evaluate multiple YOLO versions for lesion detection in mammograms. We utilize the open-source tools provided by Ultralytics. The training was performed on an Intel Xeon W-2295 with 64 GB of RAM (Intel Corp., Santa Clara, California, USA) and an NVIDIA RTX 3090 GPU (NVIDIA Corp., Santa Clara, California, USA) running Microsoft Windows version 11 (Microsoft Corp., Redmond, Washington, USA). The experiments were implemented in PyTorch 2.5.1, using the Compute Unified Device Architecture (CUDA) 11.8 and the CUDA Deep Neural Network (cuDNN) 9.1. Furthermore, these models were trained using a consistent set of hyperparameters to ensure a fair comparison across all generations. A total of 150 epochs were executed utilizing a standard learning rate of 0.01 and a batch size of 16. Key regularization techniques implemented included a momentum of 0.937 and weight decay of 0.0005 to facilitate optimized convergence and mitigate overfitting (Table 3).

A notable difference among the versions is the configuration of the YOLO augmentation environment. YOLOv5 employed six key features: image translation, image scale, image left-right flip, image mosaic, image mix-up, and image segment copy-paste. YOLOv8, YOLOv10, and YOLOv11 implemented four key features, omitting image mix-up. Table 4 outlines the augmentation settings for the YOLO models of the Ultralytics configuration.

### 3.3. Preprocessing Tasks

Image preprocessing is crucial for preparing data for model training, as it significantly affects training and validation outcomes. Given GPU constraints, data reduction is essential for optimal performance. This research incorporates two primary preprocessing steps: cropping and data augmentation. Cropping helps adjust the composition by removing unnecessary elements and focusing on the subject.

#### 3.3.1. Image Resizing and Fidelity Preservation

**Figure 6:**
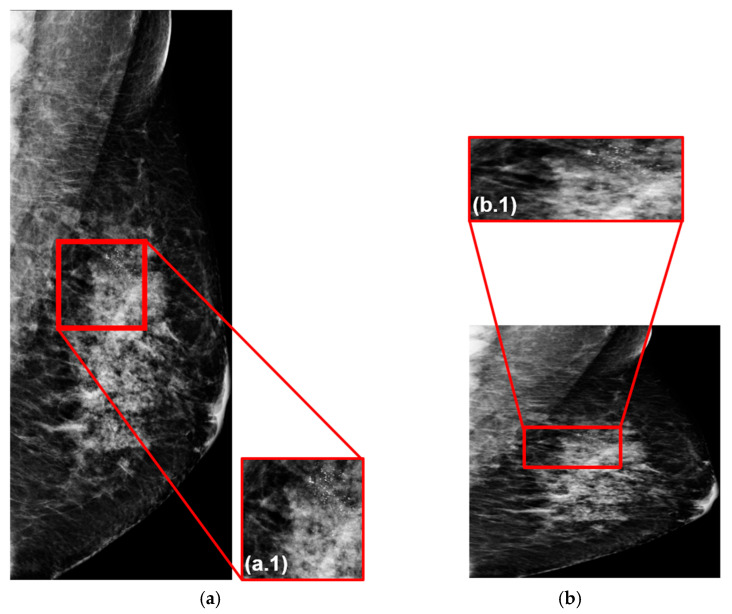
Quantitative analysis of mammogram fidelity. (**a**) Cropped mammogram (1299 × 3312 pixels) with a red bounding box indicating the ROI containing dense tissue structures (**a.1**). (**b**) Resized mammogram (640 × 640 pixels) used as input to the AI models with a red bounding box indicating the ROI containing dense tissue structures (**b.1**).

The original mammograms are in two sizes: 3328 × 4096 pixels and 2560 × 3328 pixels. To ensure compatibility with the AI model’s input layer, these images are first converted to grayscale and then thresholded to create binary images. An automated program then detects the largest contours surrounding the breasts and eliminates any no-data areas, resulting in a set of cropped mammograms with varying sizes. For example, Figure 6a shows a cropped mammogram of 1299 × 3312 pixels, which is then resized to 640 × 640 pixels for model input, as shown in Figure 6b.

To ensure that diagnostic information, particularly fine structures such as microcalcifications, is preserved after downsampling, we calculated the Structural Similarity Index (SSIM) between the cropped images (Figure 6(a.1)) and their resized counterparts (Figure 6(b.1)) [53]. The average SSIM for the training dataset was 0.828, indicating that image fidelity was highly maintained [54]. Figure 6(a.1) displays an enlarged view of the region of interest (ROI) marked in Figure 6a, and Figure 6(b.1) shows the corresponding enlarged region after resizing (from Figure 6b).

Incorporating images with varying aspect ratios during model training enhances robustness, improves generalization, and reduces geometric distortion, which helps the model recognize objects regardless of their original shape [55].

#### 3.3.2. Image Augmentation

**Figure 7:**
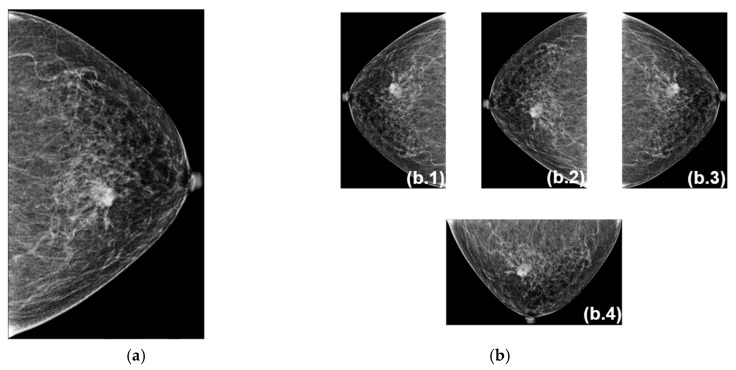
The preprocessing tasks of an image. (**a**) An original image. (**b**) Preprocessing images: (**b.1**) horizontal flip, (**b.2**) vertical flip, (**b.3**) horizontal and vertical flip, and (**b.4**) 90-degree rotation of the image.

Data augmentation includes techniques such as flipping for symmetry and rotations to enhance the dataset and reduce imbalance. During data augmentation, we generated a new set of 5148 training and 2207 validation images. The preprocessing tasks are illustrated in Figure 7. In Figure 7a, an original image is shown. In Figure 7b, the preprocessing images are a horizontal flip (b.1), a vertical flip (b.2), a horizontal and vertical flip (b.3), and a 90-degree rotation (b.4).

### 3.4. Jetson Nano Configuration

For this project, we utilized the NVIDIA Jetson Nano developer kit. The environment was configured on a compact platform featuring an NVIDIA Maxwell GPU, a Quad-Core Arm Cortex-A57 Processor, and 4 GB Low Power Double Data Rate (LPDDR) 4 Memory, using Tensor Runtime (TensorRT) Floating Point (FP) 16 precision for efficient edge inference. The software stack was built on Ubuntu 18.04, integrating PyTorch 2.5.1, CUDA 10.2, and cuDNN 8.6. Renowned for its low power consumption and cost-effectiveness, this hardware is particularly well-suited for embedded AI applications. To leverage these capabilities, we configured the Jetson Nano with a complete software stack for AI and computer vision.

### 3.5. Performance Metrics

In the experiment, we employed a suite of standard object detection metrics, including the F1-score and mean Average Precision (mAP). Fundamental to this evaluation is the Intersection over Union (IoU) score, which quantifies the extent of overlap between a predicted bounding box and its corresponding ground truth box. Based on a predefined IoU threshold, each prediction is classified as one of the following:

True Positive (TP): A real lesion (e.g., a mass or calcification) is correctly detected and classified by the model.

False Positive (FP): A normal lesion is incorrectly identified as a lesion by the model.

False Negative (FN): A real lesion is present in the image, but the model fails to detect it.

From these classifications, we calculate precision and recall by Equations (1) and (2). Precision measures the reliability of the positive predictions (i.e., of all predicted lesions), while Recall measures the model’s ability to detect all actual lesions. The formulas are as follows:

Precision = TP/(TP + FP),(1)

Recall = TP/(TP + FN).(2)

The trade-off between these two metrics across different confidence thresholds is visualized in a Precision-Recall (PR) curve. The Average Precision (AP), calculated as the area under this curve, provides a comprehensive performance summary for a single class.

### 3.6. Implementation of the Model

In this section, we explore the application of YOLO models for the automated detection of breast cancer lesions in mammography images. Our study is divided into three experimental phases:

#### 3.6.1. Initial Models Training

We evaluated multiple versions of YOLO models, including YOLOv5 (variants: v5n, v5s, v5m, v5l, and v5x), YOLOv7 (variants: v7 and v7x), YOLOv8 (variants: v8n, v8s, v8m, v8l, and v8x), YOLOv10 (variants: v10n, v10s, v10m, v10l, and v10x), YOLOv11 (variants: v11n, v11s, v11m, and v11l) and RT-DETR Large model. These models were assessed for their ability to automatically detect six lesion types: MB, MM, CB, AFB, and AFM.

#### 3.6.2. Binary Evaluation Models

We tested our models using a new, original dataset consisting of 100 patients with benign images and 100 patients with malignant images. This process involves exploring the practical application of these models in real-world clinical settings. In one patient, four images are considered. The scaled image is 640 × 640 pixels, and the AI models are used to generate a final evaluation based on accuracy, sensitivity, and specificity.

#### 3.6.3. Deploying a Mammogram on the NVIDIA Jetson Nano Board

**Figure 8:**
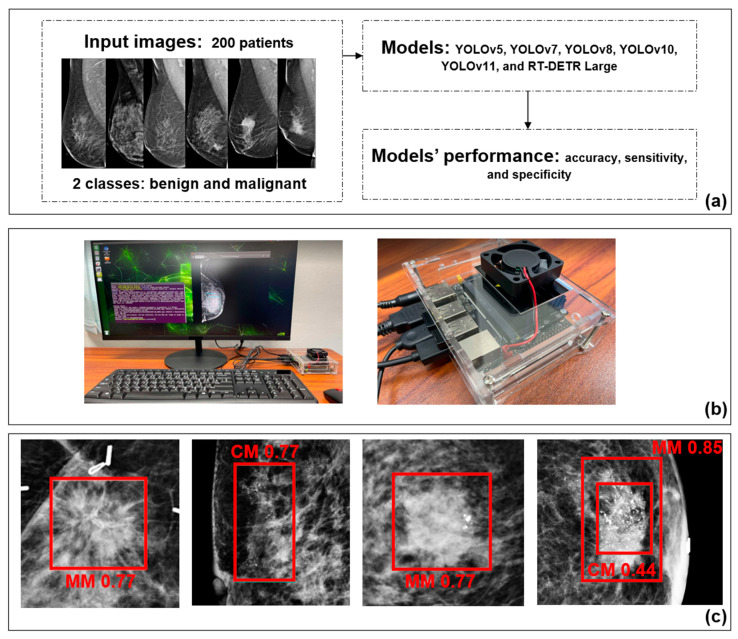
System deployment architecture on the NVIDIA Jetson Nano. (**a**) Workflow to test AI models. (**b**) NVIDIA Jetson Nano setup. (**c**) Displaying the results of lesion detection (MM: masses malignant and CM: calcification malignant).

In our process, this setup enabled us to explore the benefits of utilizing an embedded edge computing device for model inference, particularly in scenarios where real-time processing and low power consumption are crucial. For model training, we relied on a high-performance workstation with a powerful GPU, as training deep learning models requires significantly more computational resources than inference tasks. Once the models were trained, the resulting weight files were transferred to the NVIDIA Jetson Nano board for breast cancer detection tasks. This approach ensures efficient deployment, leveraging the edge device’s capabilities for real-time inference while relying on more robust systems for the computationally intensive training phase. To provide further insights, Table 5 summarizes the number of model parameters for each variant, which serves as a direct indicator of model complexity. Understanding the parameter count is crucial, as it influences both computational requirements and model performance. Additionally, Figure 8 presents the end-to-end system for real-time mammogram analysis, detailing the experimental workflow that compares multiple YOLO and RT-DETR models on a 200-patient dataset using accuracy, sensitivity, and specificity (Figure 8a). The system is deployed on the NVIDIA Jetson Nano edge device to evaluate inference speed, as shown by the hardware setup in Figure 8b. Figure 8c confirms the successful detection and classification of malignant lesions (MM and CM) with confidence scores using red bounding boxes.

## 4. Results and Discussion

### 4.1. Experiment 1: Initial Model Training

In Experiment 1, we employed multiple versions of YOLO models, ranging from YOLOv5 to the latest YOLOv11, including RT-DETR Large, to detect six lesion types in mammograms. The performance of these models is detailed in Table 6. The models were trained and evaluated using a resolution of 640 × 640 pixels, with an IoU threshold of 0.6 and a confidence threshold of 0.001. The YOLOv5m, YOLOv5l, and YOLOv5x models demonstrated exceptional performance. It achieved a mAP of 0.96. Compared to other models, YOLOv5 has the highest mAP. To further validate the performance of the YOLO models, we evaluated them across multiple public datasets. VinDr-Mammo. This dataset was used to classify lesions on mammograms as either benign or malignant. As shown in Table 7, YOLOv5n achieves the highest mAP of 0.94 on the VinDr-Mammo dataset, surpassing YOLOv8, YOLOv10, and YOLOv11.

### 4.2. Experiment 2: Model Evaluation

In Experiment 2 (Table 8), we built on the models developed from Experiment 1’s findings by selecting 200 patients to assess their performance. There are 100 patients with benign images and 100 patients with malignant images.

#### 4.2.1. Impact of Model Complexity on Generalization (Private Dataset)

A counterintuitive finding in our results is the performance disparity between lightweight models (Nano/Small) and heavier variants (Large/Extra-Large), particularly in the YOLOv8 and YOLOv11 series. For instance, YOLOv11n achieved an accuracy of 0.86, significantly outperforming YOLOv11x, which dropped to 0.75. This inverse relationship suggests that the larger model is overfitting due to the relatively small size of the medical imaging dataset compared to the Common Objects in Context (COCO) dataset. The lightweight models, having lower complexity, were compelled to learn more dominant and generalizable features, making them more robust for this specific task.

#### 4.2.2. Architectural Trade-Offs: Sensitivity vs. Specificity

When comparing across generations, YOLOv5x demonstrated the most balanced performance, securing the highest sensitivity (0.87) and accuracy (0.88). Architecturally, the backbone of YOLOv5 is a CSPDarknet-style network built from C3 blocks. In each C3 module, the input feature map is efficiently split, with one path passing through a sequence of bottleneck layers while the other is forwarded directly. This Cross-Stage Partial design, initially proposed in CSPNet, is leveraged because it demonstrably improves gradient flow and reduces redundant computation compared with conventional ResNet-style backbones [56].

The architecture further employs a Feature Pyramid Network (FPN)-PAN neck for robust multi-scale feature aggregation. Crucially, this is coupled with an anchor-based detection head that predicts class scores and bounding box offsets relative to predefined anchor boxes at each feature-map scale. Although this anchor-based detection head is architecturally older than recent anchor-free designs, its performance profile remains an attractive and highly effective baseline in the breast cancer detection task. The established anchor-based approach, which relies on generating multiple proposals, is likely advantageous on medical datasets with well-characterized object sizes and aspect ratios, directly contributing to the model’s capacity to minimize False Negatives and secure the observed high sensitivity.

In contrast, newer anchor-free architectures (YOLOv8, YOLOv10, and YOLOv11) exhibited a clear trade-off: they achieved exceptional specificity (>0.90) but at the cost of significantly reduced sensitivity (dropping to 0.50–0.60 in some cases). This indicates that the updated loss functions and assignment strategies in newer YOLO versions favor precision and are more “conservative” in their prediction strategy. Consequently, in a clinical screening context where minimizing false negatives is paramount, the anchor-based approach of YOLOv5 appears more clinically advantageous than newer, precision-oriented models.

#### 4.2.3. Role of Attention Mechanisms in the RT-DETR Model (Private Dataset)

The RT-DETR Large model provided distinct insights into the utility of Transformer-based architecture. It achieved the lowest false positive rate and a high specificity (0.95). This performance can be attributed to the self-attention mechanism, which effectively captures the image’s global context, allowing the model to distinguish background artifacts from lesions more effectively than a CNN alone. However, its moderate sensitivity suggests that, while Transformers are excellent at suppressing noise, they may require significantly larger datasets to match the lesion-detection sensitivity of CNN-based counterparts like YOLOv5.

In Experiment 2 (Table 9), we use a 10% test set of the VinDr-Mammo dataset, which is separated explicitly for testing.

#### 4.2.4. Impact of Model Complexity on Generalization (VinDr-Mammo)

The smallest model in the YOLOv5 series, YOLOv5n, achieved the highest detection performance with an mAP@0.5 of 0.92 and an F1-score of 0.87, outperforming both its larger variants and the newer architectures. The phenomenon highlights the impact of model complexity relative to dataset size. In medical imaging datasets like Vindr-Mammo, which typically have fewer samples than general datasets (e.g., COCO), heavy models (Large/Extra-large) are prone to overfitting. The lightweight YOLOv5n, with fewer parameters, demonstrated superior generalization, effectively capturing the dominant features of lesions while avoiding the background noise inherent in mammograms.

#### 4.2.5. Trade-Off Between Detection and Localization Accuracy

While the lightweight YOLOv5n model excelled in general object detection (mAP@0.5), the heavier YOLOv5x variant demonstrated superior localization precision, achieving the highest mAP@0.5:0.95 of 0.76. This suggests that although deeper networks may exhibit reduced detection recall due to overfitting on limited datasets, their complex feature-extraction layers enable more precise delineation of lesion boundaries once an object is successfully identified. This characteristic is crucial for clinical applications that require accurate measurement of tumor size.

#### 4.2.6. Role of Attention Mechanisms in the RT-DETR Model (VinDr-Mammo)

The RT-DETR Large model does not achieve the highest overall detection score (mAP) when compared to the YOLO model. The RT-DETR achieved a competitive mAP@0.5 of 0.83. The model has a moderate Recall of 0.80 and a Precision of 0.86. On this particular dataset, the RT-DETR model operates conservatively, yielding lower Sensitivity. This means the model is likely to miss a higher proportion of true lesions (false negatives) than the most sensitive YOLO variants. Due to its lower Sensitivity, the model is not recommended as the primary high-throughput screening tool.

Based on a comprehensive comparative analysis of Table 8 (private dataset) and Table 9 (VinDr-Mammo dataset), the YOLOv5 architecture demonstrated the most robust and clinically relevant performance. We recommend selecting YOLOv5n for high-throughput screening applications and YOLOv5x for high-precision diagnostic interpretation. Our findings underscore that in specialized medical imaging, robustness and controlled complexity are more valuable than mere architectural novelty.

### 4.3. Experiment 3: Deploying a Mammogram on the NVIDIA Jetson Nano Board

We deployed YOLO models for automatic detection on mammograms, utilizing two distinct hardware platforms: a GPU computer and the NVIDIA Jetson Nano Board. In the experiment, only the two highest-performing models (YOLOv5x and YOLOv11n) were chosen for subsequent performance analysis. To precisely measure inference latency, we set the batch size to 1 and fixed the input resolution at 640 × 640 pixels. For each model, we performed 10 warm-up iterations to stabilize GPU performance, then measured wall-clock time over the subsequent 50 inference runs using CUDA events.

As shown in Table 10, the inference speed results demonstrate a significant performance gap between high-end hardware and edge devices. As shown in Table 10, the inference speed results demonstrate a significant performance gap between high-end hardware and edge devices. On the RTX 3090 GPU, the lightweight YOLOv11n achieves an average inference time of 19.21 ± 0.11 ms per image (52.06 ± 0.30 FPS), making it substantially faster than YOLOv5x, which requires 66.79 ± 0.57 ms per image (14.97 ± 0.13 FPS). However, even on the resource-constrained Jetson Nano, the same trend is observed: YOLOv11n attains 162.82 ± 8.90 ms per image (6.16 ± 0.31 FPS), whereas YOLOv5x reaches only 1367.7 ± 40.95 ms per image (0.72 ± 1.55 FPS).

These results decisively demonstrate that the lightweight YOLOv11n model is considerably more suitable for real-time applications, particularly on edge devices such as Jetson Nano. Compared with YOLOv5, YOLOv11n has architectural changes. First, the FPN and PAN neck are redesigned to reduce the Floating-point Operations Per Second (FLOPS) [57], making multi-scale feature fusion more computationally efficient and directly lowering inference time. Second, an anchor-free detection head [58], in YOLOv11, simplifies the design by removing anchor boxes, reduces computational overhead, and often improves robustness to variations in object scale and aspect ratio, making it particularly attractive for real-time deployment on resource-limited edge devices. Third, the backbone, constructed from C3k2, C2f, and C2PSA modules, enhances efficiency in reducing redundant computation. Additionally, the integrated spatial attention improves feature quality by enabling the network to focus more effectively on salient regions while suppressing background noise. Collectively, these sophisticated design choices fully explain the observed improvements in speed and efficiency of YOLOv11 over YOLOv5 [8,59].

Table 11 presents the statistical analysis of GPU processing times for a computer and an NVIDIA Jetson Nano board, obtained from a test dataset comprising 100 benign and 100 malignant patients (in milliseconds). The GPU computer executed both benign and malignant workloads within the 15–17 ms range, maintaining nearly identical medians but showing moderate variance for benign patients (σ = 7.75) and tighter consistency for malignant ones (σ = 2.86). In contrast, the NVIDIA Jetson Nano showed mean processing times that were an order of magnitude higher (142–152 ms). While its medians stayed close (130.44 ms), the standard deviations were much larger (26–42 ms), suggesting that embedded GPU systems are more affected by workload spikes, thermal throttling, and memory contention. The benign Jetson runs, in particular, exhibited heavily right-skewed distributions (max = 392.85 ms) due to sporadic high-latency events. Comparing benign and malignant data across platforms reveals that malignant workloads tend to exhibit lower variance but occasionally higher peaks.

Our findings are consistent with previous studies demonstrating the advantages of YOLO-based detectors for breast lesions [42,60,61,62]. However, while primarily focused on YOLOv5, YOLOv8, or YOLOv10, our results indicate that the newer YOLOv11n architecture can provide comparable or superior accuracy at lower computational cost. For example, compared with the YOLOv5-based method proposed by [42], our model achieves a higher sensitivity at a similar false-positive rate, despite being evaluated on a more heterogeneous dataset. This suggests that modern anchor-free architecture may offer better scalability and robustness in real-world clinical settings.

From a clinical perspective, YOLOv11n’s ability to maintain high detection performance while running efficiently on low-power hardware such as the Jetson Nano is crucial for point-of-care deployment. In resource-limited settings where access to high-end workstations is limited, a lightweight model that can be embedded in existing imaging devices or used at the periphery of the hospital network could significantly increase screening throughput and reduce radiologist workload. Furthermore, the high sensitivity observed in our experiments suggests that such a system could effectively serve as a second reader, flagging suspicious regions for closer inspection rather than aiming to replace human experts entirely.

**Figure 9:**
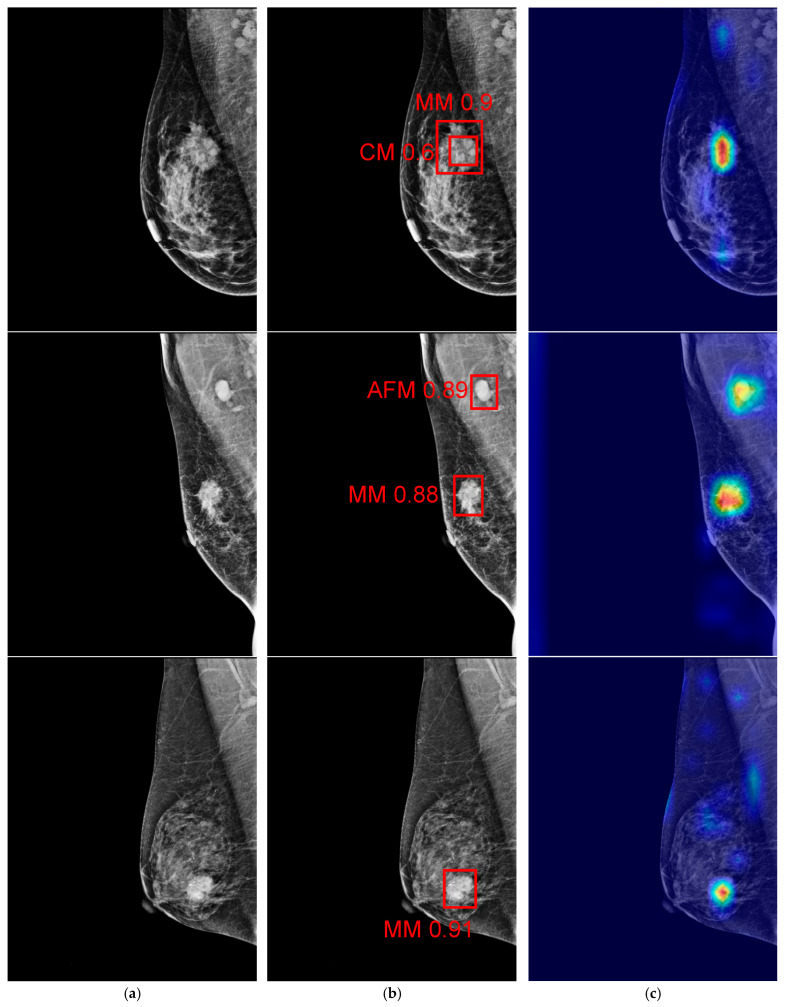
The prediction outcomes of YOLOv11n models on the NVIDIA Jetson Nano. Column (**a**): original images. Column (**b**): labelling results of the YOLOv11n model (red boxes) on original images. Column (**c**): EigenCAM images derived from labelled images in Column (**b**).

**Figure 10:**
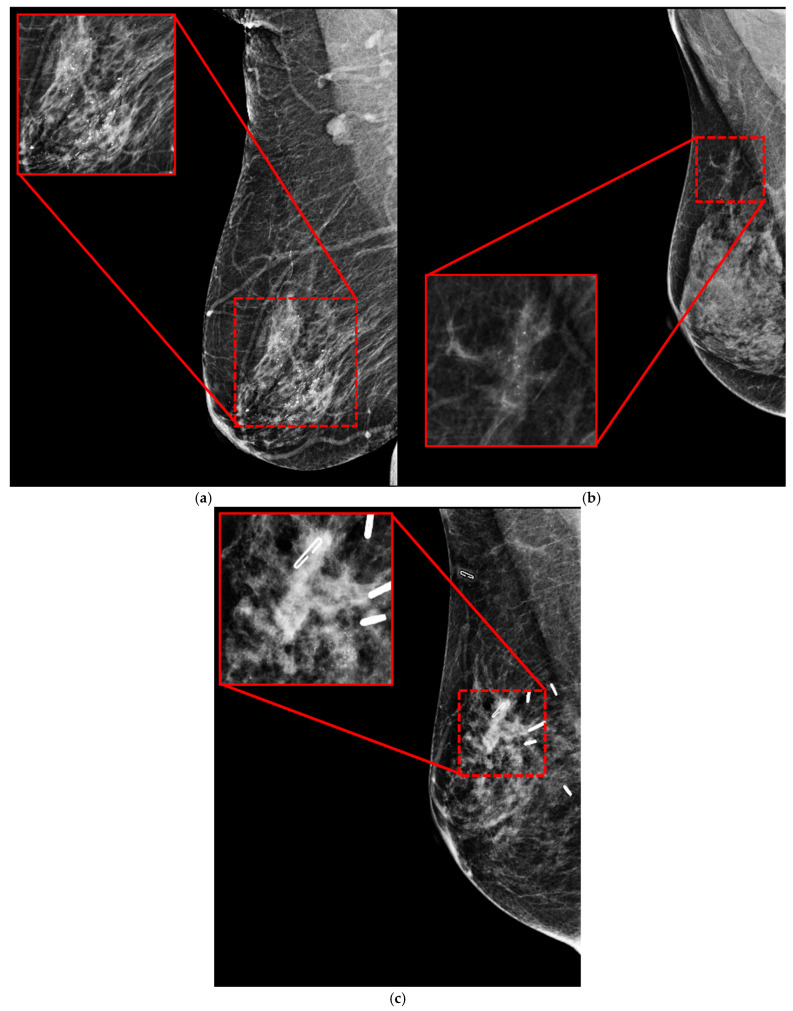
The missing outcomes of the YOLOv11n model. Column (**a**): segmental distribution of fine linear branching calcifications with diffuse large rod-like and coarse calcification in the right breast. Column (**b**): segmental, round, and short tubular microcalcification of the right breast. Column (**c**): a group of pleomorphic calcifications and segmental punctate and amorphous calcifications in the surgical area of the right breast.

Figure 9 presents the original mammograms and the prediction results of the YOLO models. In Figure 9a, the original mammogram input images are shown. Figure 9b shows detection results, including the predicted ROI (red box) and its confidence score. Figure 9c shows an Eigenvalue-based Class Activation Map (EigenCAM) [63] to highlight lesions. The hotter colors (red/orange) denote areas with the strongest influence on the prediction, effectively validating the ROI identified in column Figure 9b. Given that the YOLOv11n model is based on test results from the malignant dataset, it still fails to detect calcifications. The training dataset might not include sufficient variation in suspicious calcification morphology, which could be the cause of the issue. Figure 10 shows the results of missing objects that YOLOv11n did not detect. Figure 10a shows the segmental distribution of fine linear branching calcifications with diffuse large rod-like and coarse calcification in the right breast. Figure 10b is a segmental, round, and short tubular microcalcification of the right breast. Figure 10c shows a group of pleomorphic calcifications and segmental punctate and amorphous calcifications in the surgical area of the right breast.

## 5. Conclusions

These research findings highlight the importance of selecting appropriate YOLO models tailored to hardware constraints to achieve optimal performance in real-time applications. We compared accuracy and speed using FPS data to choose the most suitable model for use on the NVIDIA Jetson Nano, an edge computing device with limited computing resources. Therefore, the right model should strike a balance between accuracy and speed. Researchers recommended YOLOv11n for the NVIDIA Jetson Nano. It offers high accuracy (Accuracy = 0.86) and the best speed on the NVIDIA Jetson Nano (FPS = 6.16 ± 0.31). YOLOv11n stands out as the optimal choice for the Jetson Nano, offering a perfect balance of high accuracy and efficient performance, making it ideal for future use and development in edge computing applications.

In a comparative statistical analysis of the GPU computer and the NVIDIA Jetson Nano processing time, the GPU computer delivers fast, consistent real-time performance (15–17 ms), with low time variance across both benign and malignant patients. In contrast, the NVIDIA Jetson Nano operates significantly slower (142–152 ms) and suffers from substantial performance instability, especially in benign patients, where processing time spiked to 392.85 ms. This high variance on the embedded device indicates susceptibility to bottlenecks, such as thermal management and resource contention, reflecting a clear trade-off between the stable speed of a high-end GPU and the unpredictable performance of an edge-processing device.

Furthermore, there are some problems with identifying calcification abnormalities. The limited accuracy in calcification diagnosis may be due to insufficient diversity in the model’s training data. Calcifications exhibiting suspicious morphology can present in various patterns, including amorphous, coarse heterogeneous, fine pleomorphic, and delicate linear or fine-linear branching patterns. Additionally, these calcifications exhibit varying distribution patterns. Therefore, expanding the training dataset’s diversity to encompass these morphological characteristics and distribution patterns would enhance the model’s diagnostic performance for calcifications. To address this, we suggest expanding the training set to include more data and strengthening the classifier model by adding a variety of datasets to enhance its resilience. Additionally, calcifications should be identified separately from other anomaly detection because they are huge and require specialized care.

This study has several limitations. First, the dataset is derived from a limited number of scanners, which may limit the model’s generalizability to other scanners or diverse populations. Furthermore, there is no evaluation at the multiclass level on the private dataset. This multiclass analysis is clinically meaningful and should be integrated for a complete review in future work. Second, our evaluation relied exclusively on retrospective images and did not include a prospective clinical workflow or reader study; thus, the actual impact on radiologist performance and patient outcomes remains unknown. Third, we focused exclusively on two-dimensional images and did not incorporate additional clinically relevant information, such as patient age, risk factors, or multi-modality imaging, the integration of which may further improve diagnostic performance.

Future work will focus on validating the proposed model in multi-center cohorts spanning different scanners and patient populations to better assess generalizability. A prospective reader study will be necessary to quantify the system’s effects on radiologist performance, reporting time, and inter-observer variability. In addition, integrating clinical and demographic variables and combining mammography with other modalities, such as ultrasound or MRI, may further enhance overall diagnostic accuracy. Finally, incorporating explainability mechanisms and uncertainty estimates could facilitate clinician trust and support more informed decision-making.

## Figures and Tables

**Table 1:** The backbone of YOLO models.

Model	Backbone
YOLOv5	YOLOv5 CSPDarknet (CBS, C3, and SPPF)
YOLOv7	YOLOv7 (CBS, ELAN, and MP1)
YOLOv8	YOLOv8 CSPDarknet (CBS, C3, and SPPF)
YOLOv10	YOLOv10 CSPnet (C2f-Compact Inverted Block, SPPF, and PSA)
YOLOv11	YOLOv11 (CBS, C3k2, SPPF, and C2PSA)
RT-DETR	CNN-Transformer hybrid

**Table 2:** The number of lesions in the mammogram dataset.

Type of Lesion	Number of Lesions
MB	565
MM	413
CB	412
CM	143
AFB	310
AFM	115

**Table 3:** Training parameters of YOLOv5, YOLOv8, YOLOv10, and YOLOv11.

Parameter	Value
Epochs	150
Learning rate	0.01
Batch size	16
Momentum	0.937
Weight decay	0.0005

**Table 4:** Augmentation settings for YOLO models.

Augmentation Feature	v5 (Value)	v8 (Value)	v10 (Value)	v11 (Value)
Image translation	0.1	0.1	0.1	0.1
Image scale	0.9	0.5	0.5	0.5
Image flip, left and right	0.5	0.5	0.5	0.5
Image mosaic	1.0	1.0	1.0	1.0
Image mixup	0.1	-	-	-
Image segment copy-paste	0.1	-	-	-

**Table 5:** Summary of model size and computational complexity for the evaluated detectors.

Model	Number of Layers	Number of Parameters	Billions (10^9^) of Floating-Point Operations per Forward Pass (GFLOPs)
Yolov5x	498	61,992,179	154.4
Yolov8n	168	3,006,818	8.1
Yolov10s	293	8,039,604	24.5
Yolov11n	238	2,583,322	6.3
RT-DETR Large	302	31,996,070	103.5

**Table 6:** Performance of the YOLO family and RT-DETR Large on a private validation dataset.

Model	mAP@0.5	mAP@0.5:0.95	Precision	Recall	F1-Score
YOLOv5n	0.78	0.43	0.80	0.72	0.76
YOLOv5s	0.91	0.62	0.92	0.85	0.88
YOLOv5m	0.96	0.73	0.97	0.90	0.93
YOLOv5l	0.96	0.76	0.98	0.89	0.94
YOLOv5x	0.96	0.79	0.98	0.89	0.81
YOLOv7	0.85	0.88	0.75	0.59	0.77
YOLOv7x	0.69	0.42	0.73	0.63	0.67
YOLOv8n	0.86	0.60	0.87	0.80	0.83
YOLOv8s	0.94	0.74	0.96	0.88	0.91
YOLOv8m	0.92	0.76	0.95	0.85	0.90
YOLOv8l	0.90	0.74	0.91	0.80	0.86
YOLOv8x	0.91	0.77	0.95	0.83	0.89
YOLOv10n	0.83	0.54	0.83	0.75	0.79
YOLOv10s	0.75	0.47	0.78	0.67	0.72
YOLOv10m	0.68	0.44	0.72	0.63	0.67
YOLOv10l	0.65	0.43	0.76	0.59	0.66
YOLOv10x	0.77	0.57	0.91	0.69	0.78
YOLOv11n	0.80	0.52	0.82	0.73	0.77
YOLOv11s	0.89	0.62	0.90	0.81	0.85
YOLOv11m	0.87	0.63	0.90	0.78	0.84
YOLOv11l	0.90	0.68	0.94	0.79	0.86
YOLOv11x	0.91	0.71	0.93	0.83	0.89
RT-DETR Large	0.85	0.55	0.89	0.79	0.84

**Table 7:** Performance of the YOLO family and RT-DETR Large using the VinDr-Mammo validation dataset.

Model	mAP@0.5	mAP@0.5:0.95	Precision	Recall	F1-Score
YOLOv5n	0.94	0.67	0.92	0.88	0.90
YOLOv5s	0.90	0.66	0.88	0.86	0.87
YOLOv5m	0.91	0.74	0.92	0.85	0.88
YOLOv5l	0.91	0.76	0.91	0.86	0.88
YOLOv5x	0.90	0.77	0.91	0.85	0.88
YOLOv7	0.86	0.65	0.87	0.80	0.83
YOLOv7x	0.74	0.48	0.73	0.70	0.71
YOLOv8n	0.91	0.72	0.90	0.85	0.87
YOLOv8s	0.85	0.60	0.84	0.78	0.81
YOLOv8m	0.85	0.60	0.89	0.75	0.81
YOLOv8l	0.85	0.60	0.86	0.77	0.81
YOLOv8x	0.84	0.60	0.85	0.77	0.81
YOLOv10n	0.83	0.58	0.86	0.73	0.79
YOLOv10s	0.85	0.67	0.87	0.78	0.82
YOLOv10m	0.84	0.67	0.90	0.79	0.84
YOLOv10l	0.84	0.71	0.88	0.78	0.83
YOLOv10x	0.85	0.72	0.89	0.79	0.84
YOLOv11n	0.90	0.68	0.89	0.83	0.86
YOLOv11s	0.88	0.70	0.90	0.80	0.85
YOLOv11m	0.86	0.66	0.85	0.82	0.83
YOLOv11l	0.83	0.65	0.86	0.77	0.81
YOLOv11x	0.85	0.69	0.88	0.77	0.82
RT-DETR Large	0.88	0.69	0.92	0.83	0.87

**Table 8:** Performance of the YOLO family using a private dataset with 100 patients with benign images and 100 patients with malignant images.

Model	TP	TN	FP	FN	Sensitivity	Specificity	Accuracy
YOLOv5n	84	79	21	16	0.84	0.79	0.82
YOLOv5s	78	79	21	22	0.78	0.79	0.79
YOLOv5m	68	88	12	32	0.68	0.88	0.78
YOLOv5l	75	90	10	25	0.75	0.90	0.83
YOLOv5x	87	89	11	13	0.87	0.89	0.88
YOLOv8n	72	78	22	28	0.72	0.78	0.75
YOLOv8s	69	96	4	31	0.69	0.96	0.83
YOLOv8m	55	94	6	45	0.55	0.94	0.75
YOLOv8l	59	96	4	41	0.59	0.96	0.78
YOLOv8x	58	93	7	42	0.58	0.93	0.76
YOLOv10n	66	90	10	34	0.66	0.90	0.82
YOLOv10s	76	79	21	24	0.76	0.79	0.78
YOLOv10m	69	87	13	31	0.69	0.87	0.78
YOLOv10l	68	95	5	32	0.68	0.95	0.82
YOLOv10x	53	88	12	47	0.53	0.88	0.71
YOLOv11n	84	91	9	16	0.84	0.91	0.86
YOLOv11s	69	89	11	31	0.69	0.89	0.79
YOLOv11m	62	95	5	38	0.62	0.95	0.79
YOLOv11l	58	92	8	42	0.58	0.92	0.75
YOLOv11x	55	95	5	45	0.55	0.95	0.75
RT-DETR Large	67	99	1	33	0.67	0.95	0.83

**Table 9:** Performance of the YOLO family and RT-DETR Large using the VinDr-Mammo test dataset.

Model	mAP@0.5	mAP@0.5:0.95	Precision	Recall	F1-Score
YOLOv5n	0.92	0.65	0.88	0.86	0.87
YOLOv5s	0.88	0.65	0.82	0.85	0.83
YOLOv5m	0.90	0.73	0.84	0.86	0.85
YOLOv5l	0.89	0.75	0.85	0.85	0.85
YOLOv5x	0.87	0.76	0.85	0.85	0.85
YOLOv8n	0.89	0.71	0.89	0.8	0.84
YOLOv8s	0.82	0.58	0.84	0.77	0.80
YOLOv8m	0.83	0.58	0.85	0.74	0.79
YOLOv8l	0.82	0.59	0.81	0.77	0.79
YOLOv8x	0.83	0.58	0.83	0.78	0.80
YOLOv10n	0.84	0.64	0.87	0.76	0.81
YOLOv10s	0.83	0.66	0.80	0.82	0.81
YOLOv10m	0.83	0.67	0.80	0.81	0.80
YOLOv10l	0.84	0.71	0.84	0.80	0.82
YOLOv10x	0.85	0.72	0.86	0.79	0.82
YOLOv11n	0.88	0.67	0.83	0.85	0.84
YOLOv11s	0.87	0.69	0.85	0.79	0.82
YOLOv11m	0.84	0.66	0.88	0.76	0.82
YOLOv11l	0.83	0.65	0.82	0.78	0.80
YOLOv11x	0.86	0.69	0.85	0.80	0.82
RT-DETR Large	0.83	0.67	0.86	0.80	0.83

**Table 10:** Performance of the YOLO family using a private dataset on a workstation with an NVIDIA RTX 3090 GPU and the NVIDIA Jetson Nano Board. Tested with 100 patients with benign images and 100 patients with malignant images from the test set.

Platform	Model	Inference Time (ms/Image) (Average ± Std)	Frames per Second (FPS) (Average ± Std)
**RTX 3090 GPU**	YOLOv5x	66.79 ± 0.57	14.97 ± 0.13
	YOLOv11n	19.21 ± 0.11	52.06 ± 0.30
**NVIDIA Jetson Nano Board**	YOLOv5x	1367.7 ± 40.95	0.72 ± 1.55
	YOLOv11n	162.82 ± 8.90	6.16 ± 0.31

**Table 11:** Comparative statistical analysis of the NVIDIA RTX 3090 GPU of a computer and the NVIDIA Jetson Nano Processing Time. Tested with 100 patients with benign images and 100 patients with malignant images from the test set.

Metric	GPU Computer (ms)		NVIDIA Jetson Nano (ms)	
	Benign	Malignant	Benign	Malignant
Sample Size (*n*)	100 patients	100 patients	100 patients	100 patients
Mean (µ)	16.44	15.96	152.35	142.39
Median (M)	15.75	15.75	130.63	130.44
Standard deviation (*σ*)	7.75	2.86	41.62	26.57
Minimum	7.50	7.50	129.38	129.35
Maximum	30.45	46.90	392.85	256.83
First Quartile (Q1)	9.13	15.50	130.13	129.93
Third Quartile (Q3)	20.03	16.50	160.38	139.71

## Data Availability

This study uses a combination of imaging data acquired at the National Cancer Institute, Bangkok, Thailand, and Udon Thani Cancer Hospital, Udonthani, Thailand, together with imaging data obtained from publicly available databases cited in the methods in Section 3. Clinical imaging data from the National Cancer Institute and Udon Thani Cancer Hospital are not publicly available due to patient-privacy regulations and institutional data-governance policies. De-identified data products that support the main findings of this work, together with the source code, software tools, and supporting materials required to reproduce the technical pipeline, analyses, and results, are available to accredited scientific researchers from the corresponding author upon reasonable request. All requests will be considered in cooperation with the National Cancer Institute, Udon Thani Cancer Hospital, and the relevant institutional review boards and may require a data-use agreement; a response will typically be provided within 90 working days. Within these regulatory and ethical constraints, we actively encourage collaboration and will make every effort to facilitate data access for bona fide academic and AI–related research. Publicly available datasets used in this study can be accessed directly from their original repositories as referenced in the manuscript.
